# Prevalence of sensory impairments in home care and long-term care using interRAI data from across Canada

**DOI:** 10.1186/s12877-022-03671-7

**Published:** 2022-12-08

**Authors:** Dawn M. Guthrie, Nicole Williams, Atul Jaiswal, Paul Mick, Hannah M. O’Rourke, M. Kathleen Pichora-Fuller, Walter Wittich, Rinku Sutradhar

**Affiliations:** 1 Department of Kinesiology & Physical Education, Wilfrid Laurier University, Waterloo, ON Canada; 2 Department of Health Sciences, Wilfrid Laurier University, Waterloo, ON Canada; 3 School of Optometry, Université de Montréal, Montréal, Québec Canada; 4 Department of Surgery, University of Saskatchewan, Saskatoon, SK Canada; 5 Faculty of Nursing, College of Health Sciences, University of Alberta, Edmonton, AB Canada; 6 Department of Psychology, University of Toronto, Mississauga, ON Canada; 7 Institute of Health Policy, Management and Evaluation, University of Toronto, Toronto, ON Canada

**Keywords:** Sensory impairment, interRAI, Home care, Long-term care, Prevalence, Dual sensory loss, Vision loss, hearing loss

## Abstract

**Background:**

In the general population, sensory impairments increase markedly with age in adults over 60 years of age. We estimated the prevalence of hearing loss only (HL), vision loss only (VL), and a combined impairment (i.e., dual sensory loss or DSL) in Canadians receiving home care (HC) or long-term care (LTC).

**Methods:**

Annual cross-sectional analyses were conducted using data collected with one of two interRAI assessments, one used for the HC setting (*n* = 2,667,199), and one for LTC (*n* = 1,538,691). Items in the assessments were used to measure three mutually exclusive outcomes: prevalence of VL only, HL only, or DSL. Trends over time for each outcome were examined using the Cochran-Armitage trend test. A negative binomial model was used to quantify the trends over time for each outcome while adjusting for age, sex and province.

**Results:**

In HC, there was a significant trend in the rate for all three outcomes (*p* < 0.001), with a small increase (roughly 1%) each year. In HC, HL was the most prevalent sensory loss, with a rate of roughly 25% to 29%, while in LTC, DSL was the most prevalent impairment, at roughly 25% across multiple years of data. In both settings, roughly 60% of the sample was female. Males in both HC and LTC had a higher prevalence of HL compared to females, but the differences were very small (no more than 2% in any given year). The prevalence of HL differed by province after adjusting for year, age and sex. Compared to Ontario, Yukon Territory had a 26% higher rate of HL in HC (relative rate [RR] = 1.26; 95% confidence interval [CI]:1.11, 1.43), but LTC residents in Newfoundland and Labrador had a significantly *lower* rate of HL (RR: 0.57; CI: 0.43, 0.76).When combined, approximately 60% of LTC residents, or HC clients, had at least one sensory impairment.

**Conclusions:**

Sensory impairments are highly prevalent in both HC and LTC, with small sex-related differences and some variation across Canadian provinces. The interRAI assessments provide clinicians with valuable information to inform care planning and can also be used to estimate the prevalence of these impairments in specific population sub-groups.

**Supplementary Information:**

The online version contains supplementary material available at 10.1186/s12877-022-03671-7.

## Background

Sensory impairments are known risk factors for a multitude of negative outcomes [1]. For example, hearing loss (HL), on its own, is a risk factor for dementia, contributing more to the population-attributable risk than any other non-genetic risk factor [2, 3]. HL, in the absence of cognitive challenges, is associated with a faster time to admission in LTC (i.e., residential care provided in a nursing home/LTC facility) versus the presence of both HL and cognitive impairment [4]. Individuals who experience a deterioration in their hearing are more likely, than those without these changes, to have a caregiver who is distressed [5]. Individuals with a HL or a vision loss (VL) are at increased risk for difficulties with activities of daily living (ADLs; e.g., eating, bathing, dressing) and instrumental ADLs (IADLs; e.g., using the telephone, managing finances) [6–10]. VL is also associated with reduced social participation [11, 12]. Among individuals with both VL and HL, or dual sensory loss (DSL), social participation is even more restricted, and is associated with social exclusion and poor quality of life [13, 14]. In addition, older recipients of home care, who live with both DSL and cognitive challenges, are more likely to have reduced social engagement and communication challenges, when compared to individuals who had only sensory losses or only cognitive impairment [1]. In Canada, home care refers to publicly-funded in-home services from professionals such as nurses, personal support workers, physiotherapists, and occupational therapists. Despite the importance of these sensory impairments to everyday functioning and independence, some evidence suggests that they are under-detected and/or under-treated [15–17].

VL, HL and DSL are all associated with communication difficulties as well as psychological (depression, dementia), physical (falls) and social outcomes (social isolation, loneliness). Sensory impairments are highly prevalent among older adults (60 +), increase with age [18], and are expected to increase over time, mainly due to population aging [18–20] and an absolute increase in population size.

In a 2020 analysis of data on the Global Burden of Disease Injuries and Risk Factors [21] conducted to determine need for rehabilitation, sensory impairments were identified as the second greatest area of need after musculoskeletal disorders. The World Health Organization also suggests that the greatest burden of disability, among those aged 60 + , results from sensory impairments [18]. In Canada, three studies have estimated prevalence of sensory impairments using data from the Canadian Longitudinal Study on Aging (CLSA). The CLSA represents a prospective cohort of roughly 50,000 community-dwelling adults aged 45–85 at baseline. VL was defined as best corrected visual acuity of worse than 20/40 in the better eye (0.301 logMAR), with the participant wearing prescribed glasses or contact lenses. At least moderate HL, in the better ear, was defined as > 40 dB HL pure tone average. The prevalence of HL among those 55 and older is estimated to be 25%, and somewhat higher, at approximately 65%, among those aged 70 + , with rates increasing with age [22]. The prevalence of VL is roughly 6% [23], with an estimated incidence rate of 4% over three years, [24] while DSL prevalence is estimated to be 6% [22]. It is also recognized that DSL tends to increase with age [25], and is often higher among LTC residents [26].

There is limited Canadian research exploring prevalence rates of sensory impairments in HC and in LTC settings across provinces and territories. One cross-sectional study, using interRAI data from Ontario only, reported a HL prevalence of 9%, VL at 4%, and DSL at 19% among HC recipients [1]. In the same study, the estimated prevalence of HL and VL, in LTC facilities, were both 2% and DSL was 28%. In a similar European cross-sectional study, which also used interRAI assessment data, roughly two-thirds of all LTC/nursing home residents had a single sensory loss, and one-third experienced DSL [27]. Data from surveys, such as the CLSA provide valuable insight into community-dwelling adults, however, they cannot tell us about those receiving HC, who are known to be more impaired in their cognitive and physical functioning [1, 28–31], as compared to those living in the community [32, 33]. Older adults receiving HC and LTC are also under-represented in health services research in Canada, leaving an important gap in the literature.

Given the significant effects of sensory losses on function, well-being, and communication, and some of the current gaps in the literature, it is vital to understand the prevalence of these sensory issues in HC and LTC to better inform screening, interventions, and care planning. In this study, we used existing interRAI data to report on prevalence rates with a focus on HC clients and LTC residents in multiple parts of Canada. The organization known as interRAI is an international not-for-profit group of researchers, clinicians, and policy makers from roughly 35 countries. Its mandate is to develop and test standardized assessments to be used with frail and vulnerable populations. Instruments have been developed for a wide range of health and social service settings and have been designed to act as an integrated suite to allow for data sharing between settings [34]. Evidence from New Zealand suggests that the interRAI Home Care assessment identified more unmet needs than their existing comprehensive geriatric assessment [35].

The interRAI HC instrument is being used in 20 + countries around the world. It is a standardized clinical assessment used by HC clinicians in care planning and in making decisions regarding placement in LTC. This assessment is used routinely for all long-stay HC clients expected to receive at least 60 days of care [36]. The assessment is primarily used for clinical decision-making and includes roughly 300 items covering domains such as communication and sensory status, cognition, psychosocial well-being, informal and formal support services and physical functioning. Trained care coordinators (typically registered nurses) complete the assessment by speaking with the individual and their informal care providers, and the assessment can be supplemented with information from other health providers (e.g., primary care physicians) and from clinical records, as needed. The informal caregivers receive no training in regard to completing the assessment.

The main goal of this work was to report on the prevalence of HL, VL and DSL in both HC and LTC across Canada and over time. A secondary goal was to explore how these rates varied by age, sex, and province. Exploring differences due to sex is important given existing evidence that it can affect the prevalence of all of these sensory impairments [16, 22, 37–39], as well as the fact that sex may be a consideration when developing interventions and care planning.

## Methods

### Study design

This was a retrospective cross-sectional study of secondary data collected across most provinces and one territory in Canada. Data were collected using the Resident Assessment Instrument for Home Care (RAI-HC) and the Minimum Data Set 2.0 (MDS 2.0), in LTC. The RAI-HC is used across all regions of Ontario, Newfoundland and Labrador and Yukon Territory, and is used in some parts of British Columbia, Alberta, and Manitoba [40]. The assessment is completed every six to 12 months following admission to the HC program, or following a change in clinical status [41].

Similarly, the MDS 2.0 is a standardized assessment completed for LTC residents in multiple regions, including province-wide implementation in Ontario, Yukon Territory, and Newfoundland and Labrador. It is also used in some areas of Nova Scotia, Manitoba, Alberta, Saskatchewan, and British Columbia. The majority of items in these two assessments (for HC and LTC) are either very similar or identical in both the wording of the items and the response options. For both assessments, the data are routinely submitted to the Canadian Institute for Health Information (CIHI) who store and manage the data. CIHI is responsible for stripping the data of all identifiers before sharing the data with the University of Waterloo, through a data sharing agreement between CIHI and interRAI. The data are then stored on a secure server, at the University of Waterloo, and are available to authorized Canadian interRAI Fellows, students, and researchers.

### Home care sample

All RAI-HC assessments completed between 2008 and 2019 were included (*n* = 2,667,199), representing the most recent information available. In every year, an individual entered the study (as part of the denominator) if they completed at least one RAI-HC assessment in that year. If an individual had more than one assessment in a given year, the assessment where a sensory impairment was present was used for analysis. For example, if an individual had two assessments in 2008 and in their first assessment no sensory impairments were present, but a HL was present in their second assessment of that year, then the assessment where the HL was present was used for analysis. Alternatively, if an individual did not have a sensory impairment, or the sensory impairment was the same across all assessments completed in a year, then the first assessment for each individual was used.

#### LTC Sample

Similarly, all MDS 2.0 assessments completed between 2010 and 2018 were included (*n* = 1,538,691), representing the most recent information available. The same procedures were used, as described for the HC sample, to identify the individuals to be included in the analysis. In LTC, data were available as early as 2005; however, it was decided that only assessments starting in 2010 would be included, thereby capturing data with larger sample sizes from seven provinces (of the 10 Canadian provinces and three territories). Data before 2010 were only available for three provinces (Ontario, British Columbia, Nova Scotia), with low sample sizes. Data from Nova Scotia were not included in our analyses because they were not collected consistently over time. For example, out of the seven LTC homes in Nova Scotia that submitted data in 2010, only one of those homes continued to report data throughout the entire eight-year period.

#### Sensory measures

The RAI-HC and MDS 2.0 assess vision and hearing in the same manner. The presence of a HL was identified by a single item on the assessment that scores corrected hearing ability (i.e., with the use of hearing aids or other devices) from zero (no impairment) to three (highly impaired). A score of one or higher was used to indicate the presence of a HL. In any given year, if an individual experienced only HL across all their assessments in that year, then they were considered to have experienced HL only. Similarly, corrected VL was identified by a single item on the assessment that scores visual ability from zero (no impairment) to four (severely impaired). Again, a score of one or higher indicated VL, and this cohort included only those with a VL and not HL. The hearing and vision items have good test–retest reliability (hearing: kappa = 0.83; vision: kappa = 0.85) [42] and correlate well with performance-based measures of vision and hearing, in a sample of 200 older adults attending rehabilitation centres [43]. DSL was assessed using the Deafblind Severity Index, which uses the two items on the interRAI assessment that measure hearing and vision to identify individuals with at least minimal losses in both senses [42]. This resulted in three mutually-exclusive outcome measures, namely, VL only, HL only, and DSL.

#### Other measures

For each of the three outcomes, the analysis was stratified by age. Age (in years) was categorized into four groups, namely 18–64, 65–74, 75–84 years and 85 + . Although the majority of both HC clients (85.4%) and LTC residents (93.3%) were over 65 years of age, we kept any individual over 18 years of age in our analysis since we were interested in how trends in sensory impairments change over time for all adults in these two settings. We also stratified the three outcomes by sex (coded as male/female on the assessment).

#### Analysis

We first examined demographic characteristics (age, sex, province) at three time points, namely, 2008, 2012, and 2017 in the HC sample, to determine how the distribution of these characteristics may have changed over time. Even though the data contained assessments completed as recently as 2019, the data from 2017 were used for this comparison because this was the last year in which full data were available for all six provinces. Likewise, in the LTC sample, we examined the same three demographic characteristics in 2010, 2014, and 2018. The dataset included assessments up to 2019, but the data from 2018 were used because this was the last year in which the data were fully available across all seven provinces.

Trends over time were examined for each outcome (prevalence of HL only, VL only and DSL) using all of the existing data, with the Cochran-Armitage trend test. This tests assesses whether or not a series of proportions varies linearly over time [44, 45]. Poisson regression was initially used to explore each of the three outcomes over time while adjusting for age, sex and province. There was an indication that we had over dispersion in our data (deviance values > 1.0), so a negative binomial regression model was implemented to account for this. In this model, the rate is calculated as the count of individuals experiencing the outcome divided by person-years of observation. The assumption was that individuals who have an assessment, within a given year, contribute a full 12 months of observation to the denominator, which represents total person-years. The parameter estimate for year provides the slope of the rate over time (i.e., change per year). A two-tailed alpha level of 0.05 was used for all statistical analyses, which were completed using SAS software version 9.4 [46].

All methods were carried out in accordance with the Tri-Council Policy Statement on the Ethical Conduct for Research Involving Humans. The project represents secondary analysis of anonymized data. The research team is not involved in data collection and consent procedures are carried out by the clinical assessors following the respective local guidelines in their home province or territory. This project was reviewed and approved by the Research Ethics Board at Wilfrid Laurier University (REB #: 6504) and they waived the need of informed consent to participate. The study followed the STrengthening the Reporting of OBservational studies in Epidemiology (STROBE) guidelines [47].

## Results

### Key Findings in Home Care

**Fig. 1:**
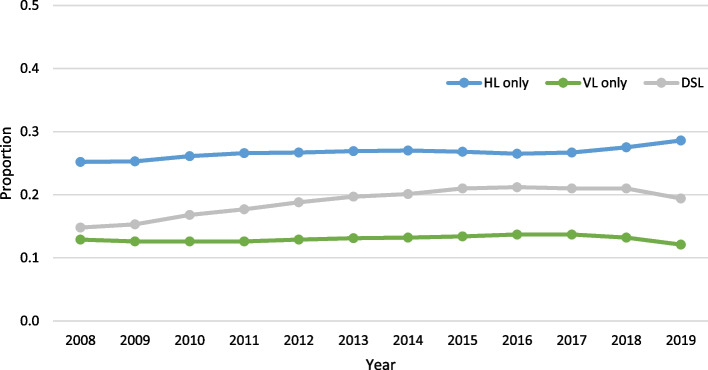
Hearing, vision and dual sensory loss (DSL) trends over time (all *p*-values < 0.001) in home care

Across the 11 years of data, each unique individual could only have one assessment for a given year, which represented a total of 2,667,199 assessments. When comparing across the three years (2008, 2012, 2017), there was an increase in the proportion who were aged 85 + (32.2% vs. 37.9% vs. 40.2%), more than half of the sample were female, and the majority of individuals were from Ontario (Table 1). There was a significant trend over time for all three outcomes (*p* < 0.001), based on the Cochran-Armitage trend test, without adjusting for any covariates. The direction of the slopes for year were all positive. However, the absolute values were extremely small, indicating a slight increase in the rate of no more than 1% per year across all three outcomes. HL was the most prevalent sensory loss, with a rate ranging from roughly 25% to 29%. DSL was the next highest, ranging from 14.8% to 21.2%, and finally, VL was roughly 12% to 14%. When looking across all three outcomes, 60% of home care clients experienced at least one of the three sensory impairments (Fig. 1).

**Table 1:** Comparison of age, sex and province distributions in the home care population in 2008 (initial year), 2012 (midpoint year), and 2017 (last year)

	2008 (*n* = 172,967)	2012 (*n* = 236,078)	2017 (*n* = 297,491)
	% (n)		
**Age (years)**			
18–64	16.8 (29,143)	14.9 (35,133)	14.0 (41,694)
65–74	14.9 (25,850)	14.2 (33,570)	15.3 (45,432)
75–84	36.0 (62,223)	33.0 (77,905)	30.5 (90,7000
85 +	32.2 (55,741)	37.9 (89,470)	40.2 (119,665)
**Sex**			
Male	34.1 (59,046)	35.7 (84,288)	37.7 (112,054)
Female	65.9 (113,921)	64.3 (151,790)	62.3 (185,437)
**Province**^**a**^			
British Columbia	7.4 (12,861)	13.9 (32,728)	12.5 (37,173)
Alberta	n/a	8.1 (19,171)	13.2 (39,108)
Manitoba	6.5 (11,183)	4.6 (10,863)	1.2 (3,429)
Ontario	86.0 (148,810)	73.3 (173,133)	70.1 (208,518)
Newfoundland & Labrador	n/a	n/a	3.0 (8,979)
Yukon Territory	0.1 (113)	0.1 (183)	0.1 (284)

^a^Provincial/territorial level data are not available across all years

**Fig. 2:**
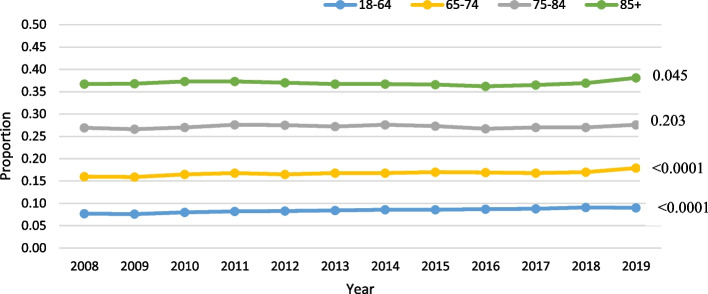
Hearing loss (HL) only trends over time by age in home care

Among those with HL, age was significant in the negative binomial model (parameter estimate for slope: 0.016; *p* < 0.0005) after adjusting for the other covariates. When HL was further stratified by specific age group, a statistically significant trend over time was found for the two youngest age groups (*p* < 0.0001), the oldest age group (*p* = 0.045), but not for the 75–84 group (*p* = 0.2). HL was most prevalent in the oldest age group (mean rate over time: 36.9%), and the least prevalent in the youngest age group (mean rate: 8.4%; Fig. 2).

**Fig. 3:**
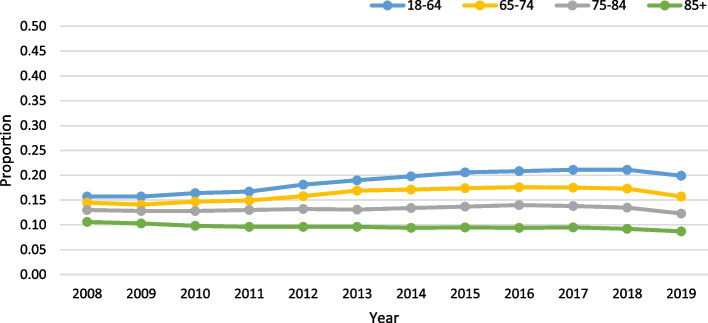
Vision loss (VL) only trends over time by age (all *p*-values < 0.0001) in home care

**Fig. 4:**
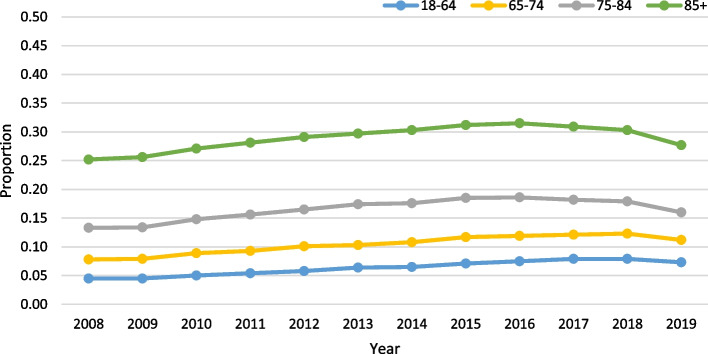
Dual sensory loss (DSL) trends over time by age (all *p*-values < 0.0001) in home care

Age was not significant, among those with VL, in the negative binomial model (slope:-0.0198; *p* = 0.35). VL was most prevalent in the 18–64 group (mean rate: 18.7%) and least prevalent in the oldest age group (mean rate: 9.6%; Fig. 3). Finally, among those with DSL, age was significant in the negative binomial model (slope: 0.0585; *p* = 0.02), with a slope indicating a roughly 6% increase per year. The rate of DSL was highest in the 85 + group at 22.6% (Fig. 4).

Among females, all three outcomes had significant trends over time based on the Cochran-Armitage test (*p* < 0.0001 in all three cases), and these findings were the same among males. In addition, males consistently had a higher prevalence of HL compared to females, with an absolute difference at each point in time of roughly 5%. Conversely, females had a slightly higher prevalence of VL compared to males, with a difference of 1–3% for each year of data reported. The prevalence of DSL was nearly identical for males and females (see Figs. 1s, 2s and 3s in Additional file 1).

In the negative binomial regression models, there were notable differences between geographic regions. For example, after adjusting for year, age and sex, individuals in Yukon Territory (compared to Ontario) had a roughly 26% *increased* rate of HL (relative rate [RR] = 1.26; 95% confidence interval [CI]:1.11, 1.43). In contrast to this, individuals in Yukon Territory had a 33% *reduced* rate of having VL (RR: 0.67; CI: 0.56, 0.8). The other factors (age, sex and year) had a minimal influence on the rates in the multivariable regression models (Table 2).

**Table 2:** Trends over time (in years) in home care after adjusting for age, province and sex from the negative binomial regression models^a^

	Hearing Loss Adjusted RR (95% CI)	*p*-value	Vision Loss Adjusted RR (95% CI)	*p*-value	Dual Sensory Loss Adjusted RR (95% CI)	*p*-value
Year	1.01 (1.00, 1.01)	0.13	1.01(1.00, 1.01)	0.12	1.01(1.00, 102)	0.06
**Province (reference = ON)**						
Alberta	1.06 (0.99, 1.14)	0.09	0.85 (0.78, 0.92)	< 0.0001	0.75 (0.67, 0.83)	< 0.0001
British Columbia	1.14 (1.02, 1.26)	0.02	0.88 (0.78, 0.99)	0.03	0.85 (0.73, 0.99)	0.03
Manitoba	1.03 (0.97, 1.09)	0.35	0.81 (0.76, 0.87)	< 0.0001	0.63 (0.58, 0.69)	< 0.0001
Newfoundland & Labrador	0.91 (0.86, 0.98)	0.01	1.12 (1.04, 1.20)	0.003	0.92 (0.83, 1.01)	0.07
Yukon Territory	1.26 (1.11, 1.43)	0.0005	0.67 (0.56, 0.80)	< 0.0001	0.95 (0.79, 1.14)	0.62
Age (in years)	1.02 (0.98, 1.05)	0.38	0.98 (0.94, 1.02)	0.35	1.06 (1.01, 1.12)	0.02
Male	1.00 (0.99, 1.01)	0.41	1.00 (0.99, 1.01)	0.77	1.00 (0.98, 1.02)	0.83

^a^*RR* relative rate, *CI* confidence interval

### Key Findings in LTC

Across the eight years of data, each unique individual could only have one assessment for a given year, which represented a total of 1,538,691 assessments. When comparing the three reference years (2010, 2014, 2018), the majority of residents were 85 + , nearly 70% were female and most were from Ontario (Table 3).

**Table 3:** Comparison of age, sex and province distributions in the LTC population in 2008 (initial year), 2012 (midpoint year), and 2017 (last year)

	2010 (*n* = 138,913)	2014 (*n* = 155,434)	2018 (*n* = 163,585)
	% (n)		
**Age (years)**			
18–64	6.5 (9,011)	6.7 (10,465)	6.8 (11,151)
65–74	9.7 (13,470)	10.5 (16,380)	11.6 (19,016)
75–84	31.7 (44,007)	28.7 (44,575)	27.5 (45,057)
85 +	52.1 (72,425)	54.1 (84, 014)	54.0 (88,361)
**Sex**			
Male	30.8 (42,778)	32.4 (50,382)	33.7 (55,139)
Female	69.2 (96,135)	67.6 (105,052)	66.3 (108,446)
**Province**			
British Columbia	17.8 (24,695)	18.4 (28,605)	18.4 (30,020)
Alberta	11.4 (15,884)	11.0 (17,107)	11.1 (18,149)
Manitoba	4.5 (6,313)	4.2 (6,586)	4.1 (6.619)
Saskatchewan	n/a	3.6 (5,595)	5.5 (9,045)
Ontario	65.8 (91,416)	61.1 (94,885)	58.9 (96,410)
Newfoundland & Labrador	0.3 (455)	1.5 (2,249)	1.9 (3,115)
Yukon Territory	0.1 (150)	0.1 (165)	0.1 (227)

**Fig. 5:**
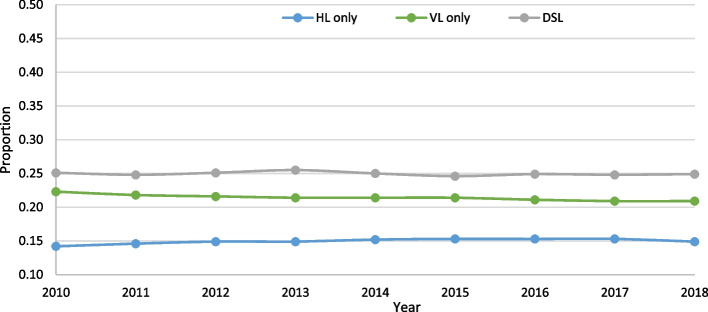
Hearing, vision and dual sensory loss (DSL) trends over time (all *p*-values < 0.001) in LTC

When analyzing all eight years of data, there was a significant trend over time for all three outcomes (*p* < 0.05). The slopes for both VL (-0.0028) and DSL (-0.0044) were negative, and represented a 0.3% to 0.4% decrease in the rate per year. HL, on the other hand, had a small but positive slope (0.0054). DSL was the most prevalent impairment, with a rate of roughly 25% across the eight years of data. VL was next highest, with a rate of approximately 22%, and HL was lowest, with a prevalence of around 15%. When combined, 61% of LTC residents had at least one sensory impairment (Fig. 5).

**Fig. 6:**
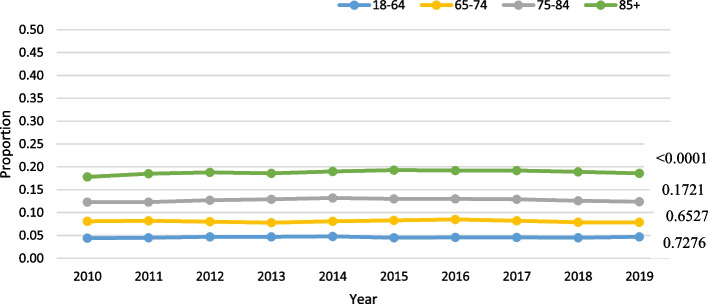
Hearing loss (HL) only trends over time by age in LTC

**Fig. 7:**
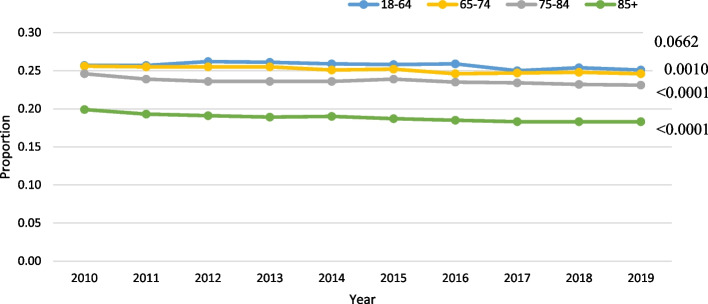
Vision loss (VL) only trends over time by age in LTC

**Fig. 8:**
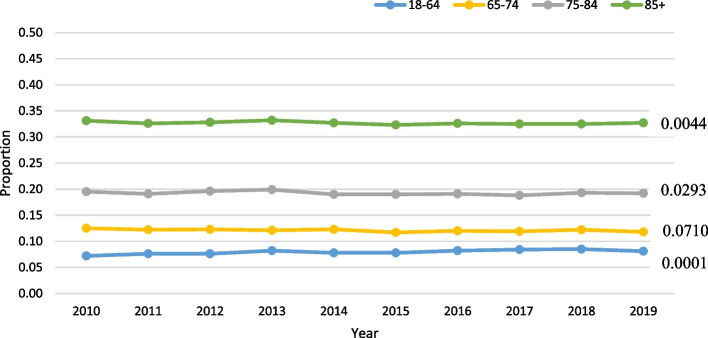
Dual sensory loss (DSL) trends over time by age in LTC

Among LTC residents with HL, age was not significant in the negative binomial model (parameter estimate for slope: 0.012; *p* = 0.79), after adjusting for the other covariates. When HL was stratified by age group, the actual rates were very stable over time across all groups (Fig. 6). Among those with VL, age was not significant in the model (slope = -0.0665; *p* = 0.24). Among the various age groups, the two youngest groups had the highest prevalence of VL, ranging from 24.6% to 26.1%, while the oldest age group (85 +) had a rate of 18–20% (Fig. 7). Similarly, among those with DSL, age was not significant in the model (slope = 0.0343; *p* = 0.47). The oldest age group (85 +) had the highest prevalence by age, at roughly 33% (Fig. 8).

There were very similar prevalence rates over time when the sensory impairments were stratified by sex. Similar to HC, males in LTC had a slightly higher prevalence of HL, but the differences were very small at no more than 2% for each year. As was seen in HC, females had a slightly *higher* prevalence of VL than males. The rates of DSL were nearly identical when stratified by sex (see Additional file 1, Figs. 4s, 5s and 6s).

As was seen in the home care sample, the negative binomial models also showed some significant associations between province and sensory impairment rates. For example, Newfoundland and Labrador stands out as residents in that province had a significantly *lower* rate, as compared to Ontario, of experiencing HL (RR: 0.57; CI: 0.43, 0.76), even after adjusting for age, sex and year. Residents in Yukon Territory had a 56% *lower* rate of experiencing VL compared to those in Ontario (RR: 0.44; CI: 0.21, 0.92). There was also a 43% reduction in the rate of experiencing DSL among those from Newfoundland and Labrador compared to Ontario (RR: 0.57; CI: 0.42, 0.78). In all three multivariable regression models, age, sex and year were not significant (Table 4).

**Table 4:** Trends over time (in years) in LTC after adjusting for age, province and sex from the negative binomial regression models

	HL RR (95% CI)	*p*-value	VL RR (95% CI)	*p*-value	DSL RR (95% CI)	*p*-value
Year	1.01 (0.98, 1.03)	0.69	1.00 (0.96, 1.03)	0.87	1.00 (0.97, 1.02)	0.76
**Province (reference = Ontario)**						
Alberta	0.75 (0.58, 0.97)	0.03	1.05 (0.75, 1.45)	0.79	1.34 (1.01, 1.77)	0.04
British Columbia	0.90 (0.72, 1.12)	0.36	1.04 (0.78, 1.38)	0.80	1.00 (0.79, 1.27)	0.98
Manitoba	1.01 (0.82, 1.24)	0.92	1.17 (0.91, 1.52)	0.23	1.21 (0.96, 1.51)	0.10
Newfoundland & Labrador	0.57 (0.43, 0.76)	< 0.0001	1.02 (0.71, 1.45)	0.94	0.57 (0.42, 0.78)	0.0004
Saskatchewan	1.16 (0.90, 1.51)	0.25	0.74 (0.53, 1.04)	0.08	0.94 (0.71, 1.25)	0.70
Yukon Territory	1.54 (0.85, 2.80)	0.16	0.44 (0.21, 0.92)	0.03	1.32 (0.70, 2.51)	0.39
Age	1.01 (0.93, 1.10)	0.79	0.94 (0.84, 1.05)	0.24	1.03 (0.94, 1.14)	0.47
Male	1.01 (0.96, 1.06)	0.65	1.00 (0.94, 1.07)	0.98	1.02 (0.97, 1.08)	0.42

## Discussion

Sensory impairments are highly prevalent among both HC recipients and LTC residents from various parts of Canada, but have remained quite stable over time. HL was the most prevalent issue in home care, affecting roughly one-quarter of individuals, whereas DSL was highest in LTC at 25%. Of particular interest is the fact that 60% of individuals, in either setting, had at least one of these sensory challenges. The high prevalence of these sensory impairments in is line with the Global Burden of Disease studies, in which sensory impairment was the second highest cause of years lived with disability globally in high-income countries [48]. To our knowledge, this is the first paper to report on the prevalence of these rates using interRAI data from multiple regions of Canada.

### Hearing loss (HL)

In the home care sample, the prevalence of HL (25–29%) is in line with previous research using RAI-HC data in Ontario[4, 49] and with data from the CLSA among community-dwelling adults aged 45–86 at baseline [22]. Likewise, the prevalence of HL in LTC (15%) is comparable to a study in Japan [50] and another in Europe [27]. However, these prevalence rates in LTC are likely under-estimated given that HL is under-detected in this setting, possibly in up to 50% of residents [17]. HL was consistently higher among males in our study, in both HC and LTC, which is supported in other literature [16, 22, 37, 38], and is often attributed to their increased exposure to occupational noise [51]. Age is a known risk factor for HL [52, 53], and we found the highest prevalence among those aged 85 + , in both settings. We also observed, in the HC sample only, an increased rate in Yukon Territory as compared to Ontario. Understanding the differences between provinces was a secondary objective of our research. As such, we did not undertake any further analyses to understand how characteristics between provinces might explain some of these differences. However, these type of analyses would be of interest to our team in future studies.

### Vision Loss (VL)

In our home care sample, the prevalence of VL (approximately 12%) was *lower* than that reported for HC clients in 12 European countries, also based on RAI-HC data. Their rates ranged from 20% (Norway) to 55% (France), using a similar definition of VL [10]. The lower prevalence in our study is likely due to the European study’s exclusion of those under 65. In contrast, our estimate was slightly *higher* than that based on data from the CLSA (at 6%), which used behavioral measures of sensory impairments [23]. This is likely due to the fact that the use of different measures of VL and HL can lead to different prevalence estimates [54].

Among the LTC residents in our sample, 22% experienced some degree of VL, nearly identical to that reported by Yamada et al. in eight European countries, which also used interRAI data [27]. Unlike HL, the prevalence of VL, in our study, was actually highest in the youngest age group (18–64), in both HC and LTC. This is likely due to the fact that the three outcomes were mutually exclusive. Since older adults are more likely to have HL as well as VL as they age, they would populate the DSL group in our sample. As a result, the decline in we see in VL is likely explained by the increasing rate of DSL with age. We found only one study comparing the prevalence of VL across Canada [23], which reported higher rates of VL in four provinces (Alberta, British Columbia, Newfoundland and Labrador, Nova Scotia), as compared to Ontario. Our analysis also showed that Newfoundland and Labrador had higher rates than Ontario, in the HC sample, but no other region showed this difference. We found nearly identical rates of VL in both home care and LTC, among females, in line with previous studies [23, 55]. VL is important to identify and correct given its link to adverse outcomes such as mortality [9, 56], reduced independence in activities of daily living [9, 12], difficulty with mobility [57], and reduced social participation [11, 12]. It should be noted that previous studies on the prevalence of VL or HL do not always exclude individuals with DSL. As a result, some of the discrepancies we see in the literature are related to the fact that those with DSL are not assigned to a unique category, as was done in the current project.

### Dual Sensory Loss (DSL)

The proportion of individuals with DSL in our HC sample (15%-21%) was nearly identical to that comparing HC clients in Canada, the US, Finland and Belgium (ranging from 13–25%), which also used a similar definition and RAI-HC data [26]. As anticipated, our rate was slightly *higher* than that based on survey data among those aged 50 + in multiple European countries (prevalence of 6%) [58]. This was expected given the older age of our sample. The prevalence of DSL in our LTC sample was very similar to that in other European countries [27] and in one study in Japan [50]. Not only was the rate of DSL high in our analysis, but it increased with age, in both settings, in line with other research [25, 26, 59]. However, we found very little difference in the prevalence of DSL between males and females, similar to other data in community-dwelling older adults [22] and those in HC or LTC [26].

DSL is considered a unique disability whereby individuals cannot accommodate for the loss in one sense by using the other sense [60]. Like single sensory impairments, it is also important to identify individuals with this impairment since it profoundly influences individuals’ abilities to gather information about their surroundings and is associated with impaired mobility [61], as well as impaired communication function and social isolation [62].

The current study utilized a very large sample size with data from multiple provinces and one territory. This work addresses an important gap in the existing home care literature, where nearly half of all previous studies in Canada were based on data from Ontario only [63]. There are several limitations, however, that should be noted. For example, the assessment of vision and hearing on the interRAI instruments are considered subjective measures of functional vision and hearing. The assessment is completed by trained professionals and involves a combination of self-report and the assessed level of impairment as judged by the individual assessor. The assessor can include information from informal care providers and standard medical tests, when they are available. Although the assessment is not an objective measure, the vision and hearing items correlate well with performance-based measures of vision and hearing [43]. The data here represent multiple provinces and Yukon Territory. However, use of the assessment varies by province, and therefore the rates are only generalizable to those regions using the particular interRAI assessment.

## Conclusions

The prevalence of sensory impairments is high among older Canadians receiving HC or living in a LTC facility. Screening for sensory impairments is an integral part of a comprehensive geriatric assessment [64]. Detecting these impairments provides an opportunity to reduce their risk on subsequent negative outcomes, such as cognitive impairment [4], and caregiver distress [5]. The onset of new sensory impairments is roughly a year among HC clients and LTC residents in Canada [65]. There is therefore an important window of opportunity for clinicians working in these two settings to screen for these impairments and implement strategies to mitigate their influence. These strategies could include a referral to audiology, optometry or speech-language pathology for further assessment and intervention. Screening and early treatment would serve to maximize the person’s ability to function using their residual vision and hearing.

The same holds true for family caregivers. Screening and early identification of sensory challenges, within the HC setting, provide an ideal opportunity for HC clinicians to intervene as soon as the impairments are recognized. Through individualized care planning, HC clinicians can make referrals to other sensory rehabilitation specialists to enable the client to receive tailored interventions, thereby remaining as independent as possible. This can then have a very positive effect on informal caregivers and could reduce their risk of caregiver distress.

Ideally, screening protocols should minimize assessment burden for health care providers and should examine vision and hearing function at the same time [66]. The interRAI assessments fulfill this need since they are routinely used in multiple parts of Canada and provide real-time information about sensory impairments without the need for additional assessment tools. As we have demonstrated, the interRAI data can be useful, at a population level, to estimate the prevalence of sensory challenges.

## Supplementary Information

**Additional file 1:** **Figure 1s.** Hearing loss (HL) only trends over time by sex (all *p*-values <0.0001) in home care. **Figure 2s.** Vision loss (VL) only trends over time by sex (all *p*-values <0.0001) in home care. **Figure 3s.** Dual sensory loss (DSL) trends over time by sex (all p-values <0.0001) in home care. **Figure 4s.** Hearing loss (HL) only trends over time by sex in LTC. **Figure 5s.**. Vision loss (VL) only trends over time by sex (both *p*-values <0.0001) in LTC. **Figure 6s.** Dual sensory loss (DSL) trends over time by sex in LTC.

## Data Availability

The datasets analyzed during the current study are not publicly available since the interRAI assessments are shared by various provinces and territories with limited access in the respective data sharing agreements. The data can be requested from the Canadian Institute for Health Information (https://www.cihi.ca/en/access-data-and-reports/make-a-data-request).

## Abbreviations

ADLs: Activities of daily living
CIHI: Canadian Institute for Health Information
CLSA: Canadian Longitudinal Study on Aging
CI: Confidence interval
HC: Home care
HL: Hearing loss
IADLs: Instrumental activities of daily living
DSL: Dual sensory loss
LTC: Long-term care
MDS 2.0: Minimum Data Set 2.0
RR: Relative rate
RAI-HC: Resident Assessment Instrument for Home Care
VL: Vision loss

## References

[1] Guthrie DM, Davidson JGS, Williams N, Campos J, Hunter K, Mick P, Orange JB, Pichora-Fuller MK, Phillips NA, Savundranayagam MY (2018). Combined impairments in vision, hearing and cognition are associated with greater levels of functional and communication difficulties than cognitive impairment alone: Analysis of interRAI data for home care and long-term care recipients in Ontario. PLoS ONE.

[2] Livingston G, Sommerlad A, Orgeta V, Costafreda SG, Huntley J, Ames D, Ballard C, Banerjee S, Burns A, Cohen-Mansfield J (2017). Dementia prevention, intervention, and care. Lancet.

[3] Livingston G, Huntley J, Sommerlad A, Ames D, Ballard C, Banerjee S, Brayne C, Burns A, Cohen-Mansfield J, Cooper C (2020). Dementia prevention, intervention, and care: 2020 report of the Lancet Commission. Lancet.

[4] Williams N, Phillips NA, Wittich W, Campos JL, Mick P, Orange JB, Pichora-Fuller MK, Savundranayagam MY, Guthrie DM (2020). Hearing and cognitive impairments increase the risk of long-term care admissions. Innov Aging.

[5] Williams N, Guthrie DM, Davidson JGS, Fisher K, Griffith LE: A deterioration in hearing is associated with functional and cognitive impairments, difficulty with communication, and greater health instability. *Journal of applied gerontology : the official journal of the Southern Gerontological Society* 2018:1–28. 10.1177/0733464818755312 29402165

[6] Choi JS, Betz J, Deal J, Contrera KJ, Genther DJ, Chen DS, Gispen FE, Lin FR: A comparison of self-report and audiometric measures of hearing and their associations with functional outcomes in older adults. *Journal of Aging and Health* 2015. 10.1177/0898264315614006 PMC5937530 26553723

[7] Slaughter SE, Hopper T, Ickert C, Erin DF (2014). Identification of hearing loss among residents with dementia: Perceptions of health care aides. Geriatr Nurs.

[8] Chen DS, Betz J, Yaffe K, Ayonayon HN, Kritchevsky S, Martin KR, Harris TB, Purchase-Helzner E, Satterfield S, Xue Q-L (2015). Association of hearing impairment with declines in physical functioning and the risk of disability in older adults. Journal of Gerontology: MEDICAL SCIENCES.

[9] Reuben DB, Mui S, Damesyn M, Moore AA, Greendale GA (1999). The prognostic value of sensory impairment in older persons. J Am Geriatr Soc.

[10] Grue EV, Finne-Soveri H, Stolee P, Poss J, Sorbye LW, Noro A, Hirdes JP, Ranhoff AH (2009). Recent visual decline - A health hazard with consequences for social life: a study of home care clients in 12 countries. Current Gerontology and Geriatrics Research.

[11] Laliberte Rudman D, Gold D, McGrath C, Zuvela B, Spafford MM, Renwick R (2016). "Why would I want to go out?": Age-related vision loss and social participation. Canadian Journal of Aging.

[12] Grue EV, Finne-Soveri H, Stolee P, Poss J, Sorbye LW, Noro A, Hirdes JP, Ranhoff AH: Recent visual decline - A health hazard with consequences for social life: a study of home care clients in 12 countries. *Current Gerontology and Geriatrics Research* 2009, 2010. 10.1155/2010/503817 PMC2929488 20811648

[13] Jaiswal A, Aldersey H, Wittich W, Mirza M, Finlayson M (2018). Participation experiences of people with deafblindness or dual sensory loss: A scoping review of global deafblind literature. PLoS ONE.

[14] Jaiswal A, Fraser S, Wittich W: Barriers and facilitators that influence social participation in older adults with dual sensory impairment. *Frontiers in Education* 2020, 5.

[15] Sinoo MM, Kort HS, Duijnstee MS (2012). Visual functioning in nursing home residents: information in client records. J Clin Nurs.

[16] Lin FR, Thorpe R, Gordon-Salant S, Ferrucci L (2011). Hearing loss prevalence and risk factors among older adults in the United States. J Gerontol A Biol Sci Med Sci.

[17] Cohen-Mansfield J, Taylor JW (2004). Hearing aid use in nursing homes, part 1: Prevalence rates of hearing impairment and hearing aid use. J Am Med Dir Assoc.

[18] World Health Organization: World Report on Ageing and Health. In*.*; 2015.

[19] World Health Organization (2018). Addressing the rising prevalence of hearing loss.

[20] Bourne RRA, Flaxman SR, Braithwaite T, Cicinelli MV, Das A, Jonas JB, Keeffe J, Kempen JH, Leasher J, Limburg H (2017). Magnitude, temporal trends, and projections of the global prevalence of blindness and distance and near vision impairment: a systematic review and meta-analysis. Lancet Glob Health.

[21] Cieza A, Causey K, Kamenov K, Hanson SW, Chatterji S, Vos T: Global estimates of the need for rehabilitation based on the Global Burden of Disease study 2019: a systematic analysis for the Global Burden of Disease Study 2019. *The Lancet* 2020. 10.1016/S0140-6736(20)32340-0 PMC7811204 33275908

[22] Mick P, Hämäläinen A, Kolisang L, Pichora-Fuller MK, Phillips N, Guthrie DM, Wittich W: The prevalence of hearing, vision and dual sensory loss in older Canadians: An analysis of data from the Canadian Longitudinal Study on Aging. *Canadian Journal on Aging* 2020, 1–22. 10.1017/S0714980820000070 32546290

[23] Aljied R, Aubin M, Buhrmann R, Sabeti S, Freeman EE: Prevalence and determinants of visual impairment in Canada: Cross-sectional data from the Canadian Longitudinal Study on Aging. *Canadian Journal of Ophthalmology* 2018. 10.1016/j.jcjo.2018.01.027 29784168

[24] Kahiel Z, Aubin MJ, Buhrmann R, Kergoat MJ, Freeman EE: Incidence of visual impairment in Canada: The Canadian Longitudinal Study on Aging. *Can J Ophthalmol* 2021. 10.1016/j.jcjo.2021.01.020 33609443

[25] Caban AJ, Lee DJ, Gomez-Marin O, Lam BL, Zheng DD (2005). Prevalence of concurrent hearing and visual impairment in US adults: the national health interview survey, 1997–2002. Am J Public Health.

[26] Guthrie DM, Declercq A, Finne-Soveri H, Fries BE, Hirdes JP (2016). The health and well-being of older adults with dual sensory impairment (DSI) in four countries. PLoS ONE.

[27] Yamada Y, Vlachova M, Richter T, Finne-Soveri H, Gindin J, van der Roest H, Denkinger MD, Bernabei R, Onder G, Topinkova E: Prevalence and correlates of hearing and visual impairments in European nursing homes: results from the SHELTER study. *Journal of American Medical Directors Association* 2014:1–6. 10.1016/j.jamda.2014.05.012 24984787

[28] Garms-Homolova V, Notthoff N, Declercq A, van der Roest HG, Onder G, Jonsson P, van Hout H (2017). Social and functional health of home care clients with different levels of cognitive impairments. Aging Ment Health.

[29] de Almeida MJ, Ces S, Vanneste D, Van Durme T, Van Audenhove C, Macq J, Fries B, Declercq A (2020). Comparing the case-mix of frail older people at home and of those being admitted into residential care: a longitudinal study. BMC Geriatr.

[30] Brown EL, McAvay G, Raue PJ, Moses S, Bruce ML (2003). Recognition of depression among elderly recipients of home care services. Psychiatr Serv.

[31] Foebel AD, van Hout HP, van der Roest HG, Topinkova E, Garms-Homolova V, Frijters D, Finne-Soveri H, Jonsson PV, Hirdes JP, Bernabei R (2015). Quality of care in European home care programs using the second generation interRAI Home Care Quality Indicators (HCQIs). BMC Geriatr.

[32] Mery G, Wodchis WP, Laporte A (2016). The determinants of the propensity to receive publicly funded home care services for the elderly in Canada: a panel two-stage residual inclusion approach. Heal Econ Rev.

[33] Raina P, Wolfson C, Kirkland S, Griffith L: The Canadian Longitudinal Study on Aging (CLSA) Report on Health and Aging in Canada. *The Canadian Longitudinal Study on Aging* 2010–2015.

[34] Hirdes JP, Ljunggren G, Morris JN, Frijters DH, Finne-Soveri H, Gray L, Bjorkgren M, Gilgen R (2008). Reliability of the interRAI suite of assessment instruments: a 12-country study of an integrated health information system. BMC Health Serv Res.

[35] Parsons M, Senior H, Mei-Hu Chen X, Jacobs S, Parsons J, Sheridan N, Kenealy T (2013). Assessment without action; a randomised evaluation of the interRAI home care compared to a national assessment tool on identification of needs and service provision for older people in New Zealand. Health Soc Care Community.

[36] Ministry of Health and Long-Term Care: Community Care Access Centres: Client Services Policy Manual. In*.* Toronto, ON.; 2007.

[37] Helzner E, Cauley J, Pratt SR, Wisniewski SR, Zmuda JM, Talbott EO, de Rekeneire N, Harris TB, Rubin SM, Simonsick EM (2005). Race and sex differences in age-related hearing loss: the health, aging and body composition study. J Am Geriatr Soc.

[38] Feder K, Michaud D, Ramage-Morin P, McNamee J, Beauregard Y (2015). Prevalence of hearing loss among Canadians aged 20 to 79: Audiometric results from the 2012/2013 Canadian Health Measures Survey. Health Rep.

[39] Rius Ulldemolins A, Benach J, Guisasola L, Artazcoz L (2019). Why are there gender inequalities in visual impairment?. Eur J Pub Health.

[40] Canadian Institute for Health Information: Profile of Clients in Home Care, 2019–2020. In*.* Ottawa, ON: Canadian Institute for Health Information; 2021.

[41] Morris JN, Bernabei R, Ikegami N, Gilgen R, Frijters D, Hirdes JP, Fries BE, Steel K, Carpenter I, DuPasquier J *et al*: RAI-Home Care (RAI-HC) Assessment Manual for Version 2.0. Washington, DC: interRAI Corporation; 1999.

[42] Dalby D, Hirdes JP, Stolee P, Strong JG, Poss J, Tjam EY, Bowman L, Ashworth M (2009). Development and psychometric properties of a standardized assessment for adults who are deaf-blind. Journal of Visual Impairment and Blindness.

[43] Urqueta Alfaro A, Guthrie DM, Phillips NA, Pichora-Fuller MK, Mick P, McGraw C, Wittich W (2019). Detection of vision and /or hearing loss using the interRAI Community Health Assessment aligns well with common behavioral vision/hearing measurements. PLoS ONE.

[44] Armitage P (1955). Tests for linear trends in proportions and frequencies. Biometrics.

[45] Cochran WG (1954). Methods for strengthing the common ?2 tests. Biometrics.

[46] SAS Institute Inc.: SAS System for Windows. In*.*, 9.4 edn. Carey, NC: SAS Institute, Inc.; 2016.

[47] von Elm E, Egger M, Altman DG, Pocock SJ, Gotzsche PC, Vandenbroucke JP (2007). Strengthening the reporting of observational studies in epidemiology (STROBE) statement: guidelines for reporting observational studies. BMJ.

[48] Vos T, Allen C, Arora M, Barber RM, Bhutta ZA, Brown A (2016). Global, regional, and national incidence, prevalence, and years lived with disability for 310 disease and injuries, 1990–2015: A systematic analysis for the Global Burden of Disease Study 2015. The Lancet.

[49] Davidson JGS, Guthrie DM (2017). Older adults with a combination of vision and hearing impairment experience higher rates of cognitive impairment, functional dependence, and worse outcomes across a set of quality indicators. J Aging Health.

[50] Mitoku K, Masaki N, Ogata Y, Okamoto K (2016). Vision and hearing impairments, cognitive impairment and mortality among long-term care recipients: a population-based cohort study. BMC Geriatr.

[51] Palmer KT, Griffin MJ, Syddall HE, Davis A, Pannett B, Coggon D (2002). Occupational exposure to noise and the attributable burden of hearing difficulties in Great Britain. Occup Environ Med.

[52] Feder K, Michaud D, Ramage-Morin P, McNamee J, Beauregard Y: Prevalence of hearing loss among Canadians aged 20 to 79: Audiometric results from the 2012/2013 Canadian Health Measures Survey. In: *Health Reports.* Edited by Canada S, vol. 26. Ottawa, ON: Statistics Canada; 2015: 18–25. 26177043

[53] Pinto JM, Kern DW, Wroblewski KE, Chen RC, Schumm LP, McClintock MK (2014). Sensory function: Insights from wave 2 of the national social life, health, and aging project. J Gerontol B Psychol Sci Soc Sci.

[54] Hamalainen A, Pichora-Fuller MK, Wittich W, Phillips NA, Mick P (2021). Self-report measures of hearing and vision in older adults participating in the Canadian Longitudinal Study of Aging are explained by behavioral sensory measures, demographic, and social factors. Ear Hear.

[55] Monaco WA, Crews JE, Nguyen ATH, Arif A (2021). Prevalence of vision loss and associations with age-related eye diseases among nursing home residents Aged >/=65 Years. J Am Med Dir Assoc.

[56] Wang JJ, Mitchell P, Simpson JM, Cumming RG, Smith W (2001). Visual impairment, age-related cataract, and mortality. Arch Ophthalmol.

[57] Wang JJ, Mitchell P, Smith W, Cumming RG, Attebo K (1999). Impact of visual impairment of use of community support services by elderly persons: The Blue Mountains Eye Study. Invest Ophthalmol Vis Sci.

[58] Viljanen A, Tormakangas T, Vestergaard S, Anderson-Ranberg K: Dual sensory loss and social participation in older Europeans. *European Journal of Aging* 2013:1–13. 10.1007/s10433-013-0291-7 PMC5549142 28804323

[59] Schneider J, Gopinath B, McMahon C, Teber E, Leeder SR, Wang JJ, Mitchell P (2012). Prevalence and 5-year incidence of dual sensory impairment in an older Australian population. Ann Epidemiol.

[60] Dammeyer J (2014). Deafblindness: A review of the literature. Scandinavian Journal of Public Health.

[61] Fletcher PC, Guthrie DM: The lived experiences of individuals with acquired deafblindness: Challenges and the future. *International Journal of Disability, Community and Rehabilitation* 2013, 12(1).

[62] Heine C, Browning CJ (2004). The communication and psychosocial perceptions of older adults with sensory loss: a qualitative study. Ageing Soc.

[63] Johnson S, Bacsu J, Abeykoon H, McIntosh T, Jeffery B, Novik N (2018). No Place Like Home: A Systematic Review of Home Care for Older Adults in Canada. Can J Aging.

[64] Elsawy B, Higgins KE (2011). The geriatric assessment. Am Fam Physician.

[65] Guthrie DM, Williams N, Campos J, Mick P, Orange JB, Pichora-Fuller MK, Savundranayagam MY, Wittich W, Phillips NA: A newly identified impairment in both vision and hearing increases the risk of deterioration in both communication and cognitive performance. *Canadian Journal on Aging* 2021:1–14. 10.1017/S0714980821000313 35859361

[66] Wittich W, Höbler F, Jarry J, McGilton KS (2018). Recommendations for successful sensory screening in older adults with dementia in long-term care: a qualitative environmental scan of Canadian specialists. BMJ Open.

