# Supplementary material for: Prevalence of sensory impairments in home care and long-term care using interRAI data from across Canada
Source: BMC Geriatr. 2022 Dec 8;22:944. doi: 10.1186/s12877-022-03671-7 (PMC9733010; doi:10.1186/s12877-022-03671-7)
Supplement: Supplementary file 1 — Additional file 1: Figure 1s. Hearing loss (HL) only trends over time by sex (all p-values <0.0001) in home care. Figure 2s. Vision loss (VL) only trends over time by sex (all p-values <0.0001) in home care. Figure 3s. Dual sensory loss (DSL) trends over time by sex (all p-values <0.0001) in home care. Figure 4s. Hearing loss (HL) only trends over time by sex in LTC. Figure 5s.. Vision loss (VL) only trends over time by sex (both p-values <0.0001) in LTC. Figure 6s. Dual sensory loss (DSL) trends over time by sex in LTC. [file 12877_2022_3671_MOESM1_ESM.docx]

**Additional File**

Figure 1s. Hearing loss (HL) only trends over time by sex (all p-values <0.0001) in **home care**

Figure 2s. Vision loss (VL) only trends over time by sex (all p-values <0.0001) in **home care**

Figure 3s. Dual sensory loss (DSL) trends over time by sex (all p-values <0.0001) in **home care**

Figure 4s. Hearing loss (HL) only trends over time by sex in **LTC**

Figure 5s. Vision loss (VL) only trends over time by sex (both p-values <0.0001) in **LTC**

Figure 6s. Dual sensory loss (DSL) trends over time by sex in **LTC**
